# Heterogeneity in PD-L1 expression between primary and metastatic lymph nodes: a predictor of EGFR-TKI therapy response in non-small cell lung cancer

**DOI:** 10.1186/s12931-024-02858-3

**Published:** 2024-06-05

**Authors:** Yaohua Hu, Yidan Zhang, You Lu, Yingqi Xu, Jianlin Xu, Hua Zhong, Lei Cheng, Runbo Zhong

**Affiliations:** 1 Department of Respiratory and Critical Care Medicine, Shanghai Chest Hospital, Shanghai Jiao Tong University School of Medicine, Huaihai West Road No.241, Shanghai, 200030 China; 2 Department of Respiratory Medicine, Shanghai Tenth People’s Hospital, Tongji University School of Medicine, Yanchang Middle Road No.301, Shanghai, 200072 China

**Keywords:** NSCLC, EGFR, TKI, PD-L1, Heterogeneity

## Abstract

**Background:**

There is inconclusive evidence to suggest that the expression of programmed cell death ligand 1 (PD-L1) is a putative predictor of response to EGFR-TKI therapy in advanced EGFR-mutant non-small cell lung cancer (NSCLC). We evaluated the heterogeneity in PD-L1 expression in the primary lung site and metastatic lymph nodes to analyze the association between PD-L1 expression and response for patients treated with EGFR-TKI.

**Methods:**

This study reviewed 184 advanced NSCLC patients with EGFR mutations who received first-generation EGFR-TKI as first-line treatment from 2020 to 2021 at Shanghai Chest Hospital. The patients were divided into the primary lung site group (*n* = 100) and the metastatic lymph nodes group (*n* = 84) according to the biopsy site. The patients in each group were divided into TPS < 1%, TPS 1–49%, and TPS ≥ 50% groups according to PD-L1 expression.

**Results:**

The median PFS was 7 (95% CI: 5.7–8.3) months, and the median OS was 26 (95% CI: 23.5–28.5) months for all patients. No correlation existed between PFS or OS and PD-L1 expression. The median PFS in the primary lung site group was 11 months (95% CI: 9.6–12.4) in the TPS < 1% group, 8 months (95% CI: 6.6–9.4) in TPS 1–49% group, and 4 months (95% CI: 3.2–4.8) in TPS ≥ 50% group, with statistically significant differences (*p* = 0.000). The median OS of the TPS < 1% group and TPS ≥ 50% group showed a statistically significant difference (*p* = 0.008) in the primary lung site group. In contrast, PD-L1 expression in the lymph nodes of EGFR-mutant patients was unrelated to PFS or OS after EGFR-TKI therapy.

**Conclusion:**

PD-L1 expression from the primary lung site might predict clinical benefit from EGFR-TKI, whereas PD-L1 from metastatic lymph nodes did not.

**Trial registration:**

: This retrospective study was approved by the Ethics Committee of Shanghai Chest Hospital (ID: IS23060) and performed following the Helsinki Declaration of 1964 (revised 2008).

**Supplementary Information:**

The online version contains supplementary material available at 10.1186/s12931-024-02858-3.

## Introduction

Non-small cell lung cancer (NSCLC) is one of the most common causes of cancer death worldwide [1]. Lung adenocarcinoma (LUAD), a category of NSCLC, is the most common histological type of lung cancer. Approximately 11% of Caucasian patients and 50% of Asian patients with LUAD harbor epidermal growth factor receptor (EGFR) mutations, mainly comprising a substitution at position 858 (21L858R) and deletion mutants in exon 19 (19del) [2–4]. All three generations of EGFR-tyrosine kinase inhibitors (TKIs) can be employed as first-line therapeutic regimens for advanced NSCLC patients with EGFR mutations [5]. However, approximately 10–20% of patients exhibit primary resistance to EGFR-TKIs [6, 7], and the PFS benefit varies widely among patients receiving TKI therapy [8, 9]. Possible reasons for the suboptimal efficacy of TKI as first-line therapy in some EGFR-mutant patients include the presence of co-mutation [10], activation of the Hippo/YAP signaling pathway [11], and BIM deletion polymorphism [12], and others. In addition, the association between programmed cell death ligand 1 (PD-L1) expression in tumor cells and the efficacy of EGFR-TKIs has been a major concern recently.

PD-L1 expression status has emerged as a putative predictive marker of response to PD-1/PD-L1 therapies in patients with driver-negative mutations [13]. However, whether PD-L1 expression in patients with EGFR mutations can predict the efficacy of EGFR-TKIs is controversial. Some studies have shown that increased PD-L1 expression in EGFR-mutant NSCLC predicts primary resistance to EGFR-TKIs [7, 14–18]. However, some studies have reached the opposite conclusion [19–21]. The reason for these incongruent results may be the heterogeneity in PD-L1 expression.

PD-L1 expression may differ before and after treatment of the same cancer lesion. This difference also exists in primary and metastases from different sites [22]. A previous study found a high rate of discordance (> 80%) between the lung primary and distant metastases in NSCLC [23]. The samples obtained from metastatic lymph nodes, pleural fluid, and adrenal glands expressed higher PD-L1 than samples obtained from the primary site, while those from the liver, brain, and bone displayed lower PD-L1 expression [24]. However, there is little data regarding the association between spatial heterogeneity of PD-L1 expression and survival in EGFR-mutant NSCLC patients treated with EGFR-TKI therapy. In this study, we investigated the spatial heterogeneity of PD-L1 (between lung primary and metastatic lymph nodes) and the predictive value of PD-L1 expression for treating advanced EGFR-mutant NSCLC with EGFR-TKI.

## Materials and methods

### Patients

**Fig. 1:**
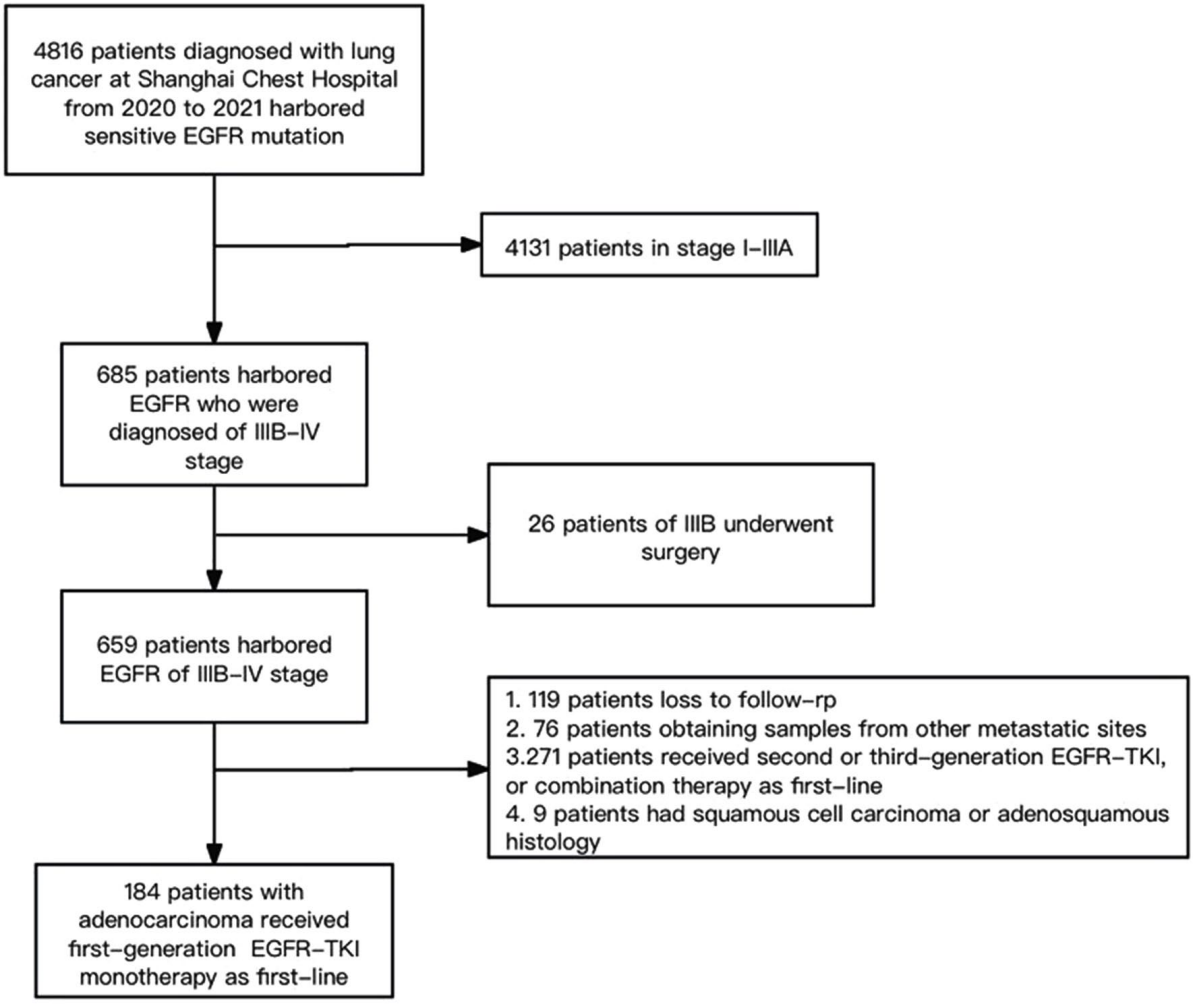
Flow chart of enrolled patients

This study reviewed the medical histories of 184 lung cancer patients treated with first-generation EGFR-TKI as first-line at Shanghai Chest Hospital from 2020 to 2021. The inclusion criteria included (I) patients with histopathologically confirmed stage IIIB-IV NSCLC according to the eighth edition of the tumor, node, metastasis (TNM) classification; (II) sampling sites including primary lung site and metastatic lymph nodes; (III) patients harboring EGFR mutations, including 19 del and 21 L858 mutations; (IV) PD-L1 detection from the samplings at diagnosis; (V) first-generation EGFR-TKI monotherapy as first-line treatment. The exclusion criteria were as follows: patients with other malignant tumors, treated with second- or third-generation EGFR-TKIs, patients who received a combination of targeted therapies, patients obtaining samples from other metastatic sites, and those without complete information or loss to follow-up. We excluded patients with squamous cell carcinoma and adenosquamous carcinoma, as they exhibit distinct biological characteristics compared to those with adenocarcinoma. (Fig. 1)

This retrospective study was approved by the Ethics Committee of Shanghai Chest Hospital (ID: IS23060) and performed following the Helsinki Declaration of 1964 (revised 2008). Informed consent was not required of the patients.

### Clinical assessments

All patients were staged according to the eighth edition of the tumor, node, and metastasis (TNM) classification before EGFR-TKI was administered as the first-line treatment. During the therapeutic course, treatment efficacy was assessed by chest computed tomography (CT) scan every 2–3 months, with additional abdominal ultrasound and cranial magnetic resonance imaging (MRI) and bone emission computed tomography (ECT), if necessary, until disease progression or termination of the therapy or the last follow-up visit, whichever occurred first. According to the Response Evaluation Criteria in Solid Tumors (RECIST, version 1.1), the response to treatment was classified as complete response (CR), partial response (PR), stable disease (SD), and stable disease (SD). The main assessment indicators included progression-free survival (PFS), overall survival (OS), objective response rate (ORR), and disease control rate (DCR). The last follow-up was conducted on June 30, 2023.

### Gene testing and PD-L1 expression tumor proportion score (TPS)

All patients underwent a tissue biopsy at the time of diagnosis. Gene testing was performed using an amplification refractory mutation system (ARMS) or next-generation sequencing (NGS). EGFR mutation types were recorded. The PD-L1 Tumor Cell proportion Score (TPS) (DACO PD-L1 IHC 22C3 pharmDx) of the tumor cells was evaluated and categorized as TPS < 1%, TPS 1–49% and TPS ≥ 50%.

### Statistical analysis

All statistical analyses were performed using SPSS 24.0 statistical software (IBM, Armonk, NY). PFS was calculated from the beginning of TKI administration to disease progression, regimen change, or last follow-up, depending on the first occurrence. OS was calculated from the beginning of TKI administration to death or last follow-up. ORR was the sum of the proportions of CR and PR, whereas DCR was the sum of the proportions of CR, PR, and SD. One-way ANOVA was used to compare the baseline characteristics of the primary lesion group and metastatic lymph node group for continuous variables and Pearson χ^2^ or Fisher exact test for categorical variables. The Kaplan-Meier method was employed to obtain the median PFS and median OS. Multivariate Cox regression was used to identify the correlation between TPS at different sites and PFS and OS after receiving first-generation EGFR-TKI. A P-value < 0.05 (two-sided) was statistically significant.

## Results

### Patient characteristics

In total, 184 patients with advanced EGFR-mutated NSCLC were included in the final analysis, and their characteristics are revealed in Table 1. The median age of the patients was 59 years old, of which 130 (70.7%) were females. There were 138 non-smokers (75.0%). Stage IIIB accounted for 20.1%, stage IIIC for 15.8%, and stage IV for 64.1%. Typically, all the patients had adenocarcinoma. Brain metastases accounted for 13.6%, bone metastases for 26.1%, and liver metastases for 6.5% at the time of diagnosis. EGFR 19Del accounted for 54.9%, and EGFR 21 L858 mutation accounted for 45.1%.

**Table 1:** Characteristics of all patients

Characteristics	Patients
Age (median)	59.0
**Gender (n, %)**	
Male	54 (29.3%)
Female	130(70.7%)
**Smoking history (n, %)**	
Smoker	46(25.0%)
Non-smoker Pathologic diagnostic process (n, %)	138(75.0%)
Biopsy of supraclavicular lymph node	31(16.8%)
TBB/TBLB	64(34.8%)
TBNA	53(28.8%)
Percutaneous lung biopsy	36(19.6%)
**TNM stage (n, %)**	
IIIB	37(20.1%)
IIIC	29(15.8%)
IV	118(64.1%)
Brain metastasis (n, %)	25(13.6%)
Bone metastasis (n, %)	48(26.1%)
Liver metastasis (n, %)	12(6.5%)
**EGFR mutation (n, %)**	
19Del	101(54.9%)
21L858	83(45.1%)
**Acquired T790M (n, %)**	
Acquired T790M	50(27.2%)
T790M negative	32(17.4%)
Not available	102(55.4%)
**PD-L1 expression (n, %)**	
< 1%	104(56.5%)
1 ∼ 49%	37(20.1%)
≥ 50%	43(23.4%)
**First-generation EGFR-TKI**	
Gefitinib	84(45.6%)
Erlotinib	50(27.2%)
Icotinib	50(27.2%)
**Best prognosis**	
PR	140(76.1%)
SD	28(15.2%)
PD	16(8.7%)

EGFR, Epidermal growth factor receptor; TBB, Transbronchial biopsy; TBLB, Transbronchial lung biopsy; TBNA, Transbronchial needle aspiration; PR, Partial response; SD, Stable disease; PD, Progressive disease

### Efficacy

The median follow-up time was 22 months. At the cutoff of follow-up, 151 patients presented disease progression after first-generation TKI therapy, and 78 died. The median PFS was 7 months (95% CI: 5.7–8.3), and the median OS was 26 months (95% CI: 23.5–28.5). The ORR of the first-line therapy was 76.1%, with a DCR of 91.3%. When EGFR-TKI therapy failed, 82 patients underwent tissue re-biopsy and gene testing, 61.0% of whom had the T790M mutations.

**Fig. 2:**
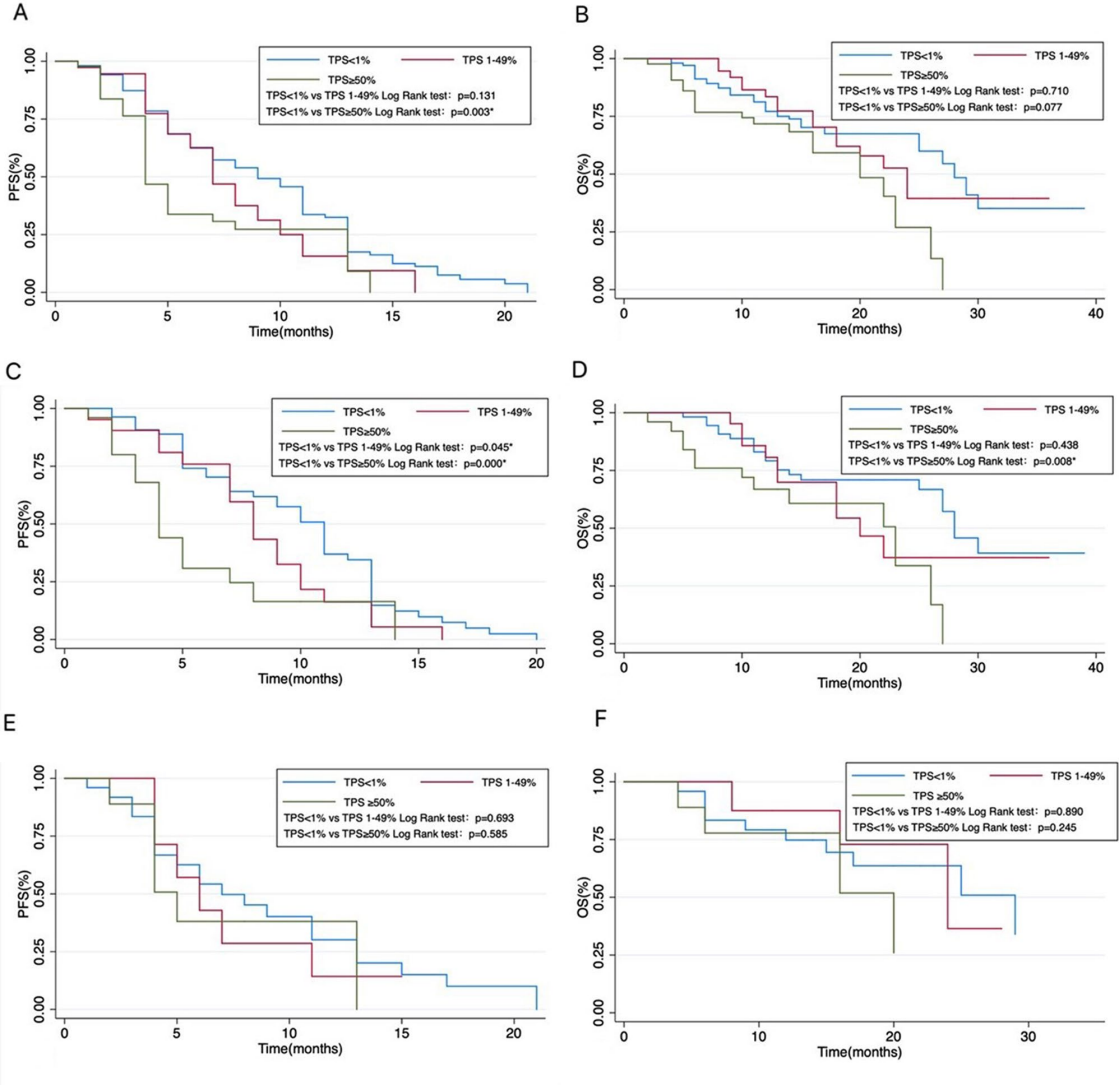
Survival curves of OS and PFS. (**A**) PFS of all patients. The median PFS was statistically significantly different between the TPS < 1% group and the TPS ≥ 50% group. (**B**) OS of all patients. No statistically significant difference was found among the three groups. (**C**) PFS in the primary lung site group. The median PFS was statistically significantly different among the three groups. (**D**) OS in the primary lung site group. The median OS was statistically significantly different between the TPS < 1% group and the TPS ≥ 50% group. (**E**) PFS in the lymph nodes group. No statistically significant difference was observed among the three groups. (**F**) OS in the lymph nodes group. No statistically significant difference was observed among the three groups. *statistical significance at *p* < 0.05. TPS, tumor cell proportion score; PFS: progression-free survival; OS: overall survival. We analyzed data from 271 excluded patients, including 31 who received first-line second-generation EGFR-TKI therapy, either as monotherapy or in combination. Another 139 patients underwent first-generation combination therapy with various agents like chemotherapy and Avastin. The remaining 101 patients received third-generation TKI therapy, including osimertinib, amatinib monotherapy, and other investigational third-generation TKIs. Among the 101, 68 received monotherapy outside clinical trials. Of these, 20 were lost to follow-up, leaving 48 for further analysis, with only 2 showing no disease progression. Thus, the number of patients on second or third-generation EGFR-TKI monotherapy was too small for group analysis compared to those on first-generation EGFR-TKI. Further study is needed on the impact of PD-L1 expression levels in the primary lung site on third-generation drugs like osimertinib

**Fig. 3:**
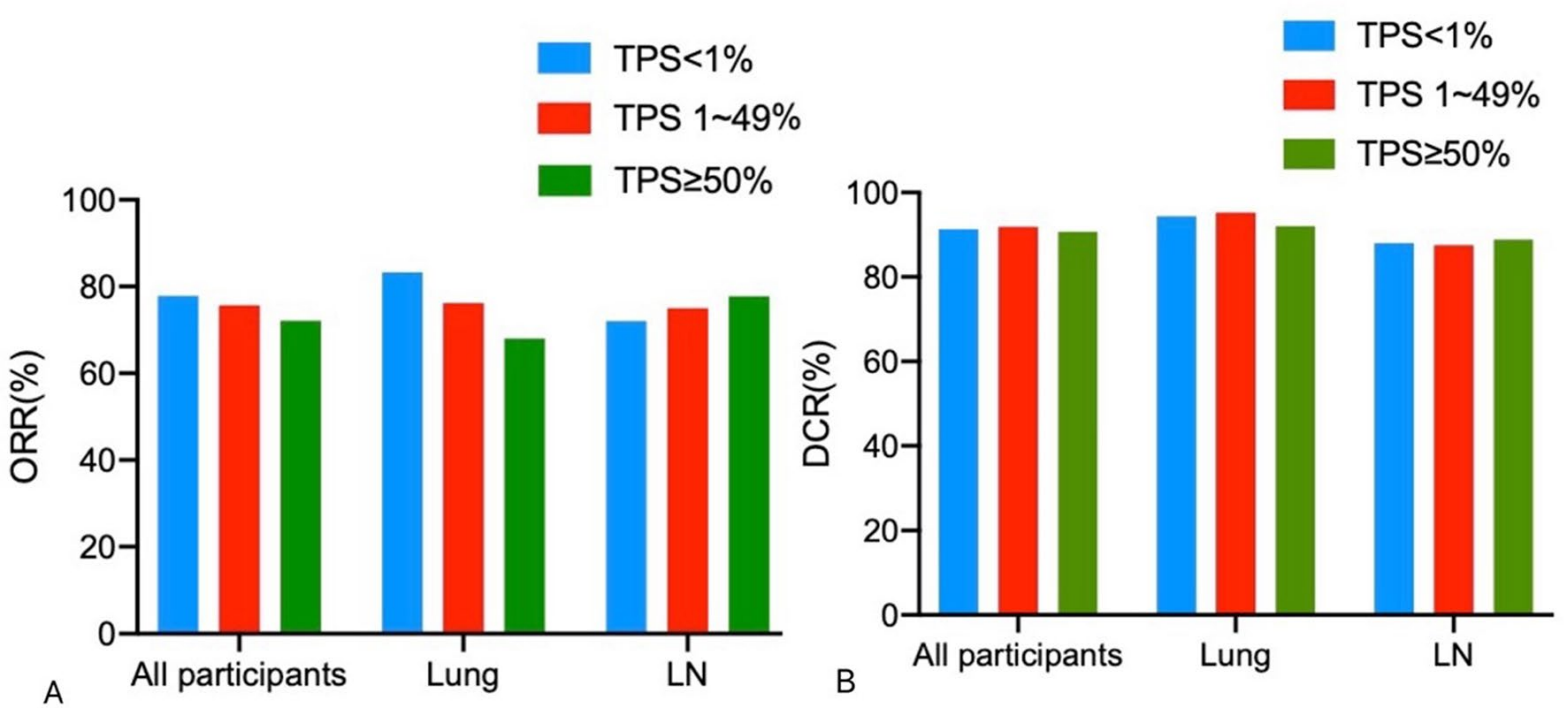
The response rate of different groups. (**A**) ORR of patients in the primary lung site and lymph node groups. No statistically significant difference was observed among the three groups. (**B**) DCR of patients in the primary lung site and lymph node groups. No statistically significant difference was observed among the three groups. TPS, Tumor cell proportion score; ORR, Objective Response Rate; DCR, Disease Control Rate; LN, Lymph node

According to the immunohistochemical detection of PD-L1 expression in tumor tissues at diagnosis, patients were divided into three groups: TPS < 1%, TPS 1–49%, and TPS ≥ 50%, of which 104 patients with TPS < 1%, 37 patients with TPS 1–49% and 43 patients with TPS ≥ 50%. The median PFS was 9 months (95% CI: 7.3–10.7) in the TPS < 1% group, 7 months (95% CI: 5.6–8.4) in the TPS 1–49% group, and 4 months (95% CI: 3.3–4.7) in the TPS ≥ 50% group, of which the median PFS was statistically significantly different between TPS < 1% and TPS ≥ 50% groups (*p* = 0.003) as illustrated in Fig. 2A. The median OS of the three groups was 28 months (95% CI: 25.0–31.0) in the TPS < 1% group, 24 months (95% CI: 19.4–28.6) in the TPS 1–49% group, and 20 months (95% CI: 15.8–27.2) in the TPS ≥ 50% group, and no significant difference existed among the three groups, as depicted in Fig. 2B. Age, gender, EGFR mutation status, and smoking history were not significantly associated with the median PFS and OS. Bone metastases were associated with a shorter OS (HR 1.996, 95% CI: 1.141–3.697, *p* = 0.015), as displayed in Fig. 3.

### Characteristics between the primary lung site group and the lymph node group

According to different sampling sites, all patients were divided into the primary lung site (*n* = 100) and lymph node (*n* = 84) groups. No statistical differences were observed in the general conditions of patients between the two groups, including the proportions of distant metastases, TNM staging, EGFR genotyping, PS scores, and TPS proportions. A comparison of the two groups is presented in Table 2.

**Table 2:** Characteristics of patients in different groups

Characteristics	Primary lung group (*n* = 100)	Lymph nodes group (*n* = 84)	*p*
Age (median)	59.0	59.0	0.853
**Gender (n,** %**)**			0.531
Male	27 (27.0%)	27 (32.1%)	
Female	73(73.0%)	57(67.9%)	
**Smoking history (n,** %**)**			0.305
Smoker	22 (22.0%)	24(28.6%)	
Non-smoker	78(78.0%)	60(71.4%)	
**TNM stage (n,** %**)**			0.840
IIIB	19(19.0%)	18(21.4%)	
IIIC	17(17.0%)	12(14.3%)	
IV	64(64.0%)	54(64.3%)	
**Brain metastasis (n,** %**)**			0.542
Brain metastasis	15(15.0%)	10(11.9%)	
No brain metastasis	85(85.0%)	74(88.1%)	
**Bone metastasis (n,** %**)**			0. 482
Bone metastasis	24(24.0%)	24(28.6%)	
No bone metastasis	76(76.0%)	60(71.4%)	
**Liver metastasis (n,** %**)**			0.754
Liver metastasis	6(6.0%)	6(7.1%)	
No liver metastasis	94(94.0%)	78(92.9%)	
**EGFR mutation (n,** %**)**			0.663
19Del	57(57.0%)	44(52.4%)	
21L858	43(43.0%)	40(47.6%)	
**TPS (n,** %**)**			0.747
< 1%	54(54.0%)	50(59.5%)	
1 ∼ 49%	21(21.0%)	16(19.1%)	
≥ 50%	25(25.0%)	18(21.4%)	
**PS score (n,** %**)**			0.205
PS = 0	6(6.0%)	2(2.4%)	
PS = 1	88(88.0%)	80(95.2%)	
PS = 2	6(6.0%)	2(2.4%)	

TPS, tumor cell proportion score; PS, performance status

### PD-L1 in the primary site of EGFR-mutant patients was associated with lower PFS and OS with EGFR-TKI therapy

**Fig. 4:**
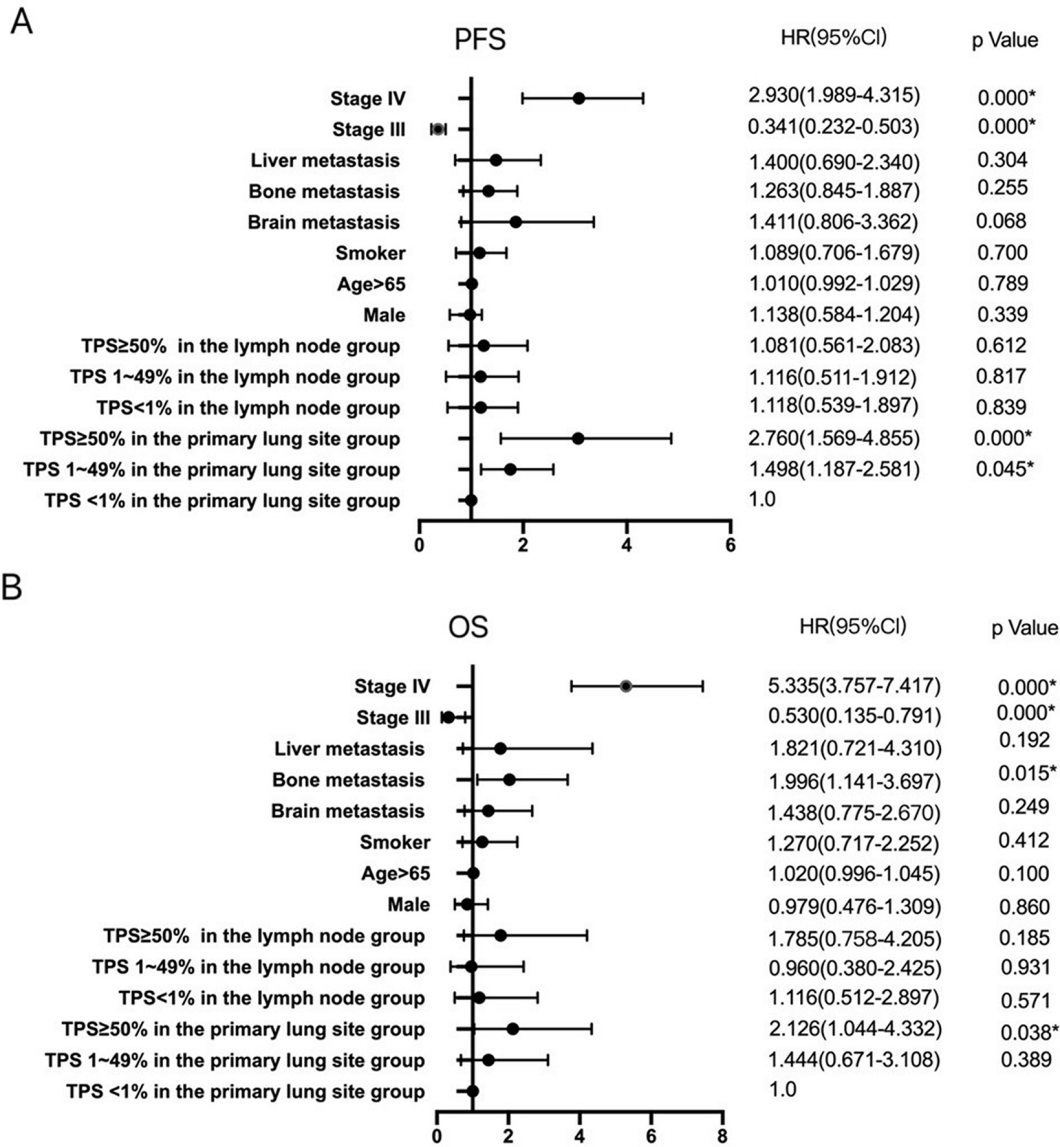
Multivariate Cox regression related to PFS and OS. (**A**) Multivariate Cox regression related to PFS. (**B**) Multivariate Cox regression related to OS. *statistical significance at *p* < 0.05. TPS, Tumor cell proportion score; PFS: Progression-free survival; OS: Overall survival

According to PD-L1 expression of the primary lung site, 100 patients were divided into three groups: TPS < 1% group, TPS 1–49% group, and TPS ≥ 50% group. The characteristics of the patients are displayed in Table 3. The ORR of the three groups was 83.3% in the TPS < 1% group, 76.2% in the TPS 1–49% group, and 68.0% in the TPS ≥ 50% group. The DCR among the three groups was 94.4% in the TPS < 1% group, 95.2% in the TPS 1–49% group, and 92.0% in the TPS ≥ 50% group. No statistical difference was identified in ORR(*p* = 0.302) and DCR (*p* = 0.881) among the three groups (Fig. 4).

**Table 3:** Characteristics of patients in the primary lung site group

	TPS < 1% (*n* = 54)	TPS 1 ∼ 49% (*n* = 21)	TPS ≥ 50% (*n* = 25)	*p*
Age (median)	54.0	59.0	59.0	0.029*
**Smoking history (**%**)**				0.391
Smoker	18.5%	33.3%	20.0%	
Non-smoker	81.5%	66.7%	80.0%	
**EGFR mutation (**%**)**				0.416
19Del	61.1%	52.4%	56.0%	
21L858	38.9%	47.6%	44.0%	
**TNM stage (**%**)**				0.907
IIIB	20.4%	14.3%	20.0%	
IIIC	18.5%	19.0%	12.0%	
IV	61.1%	66.7%	68.0%	
**Brain metastasis (**%**)**				0.327
Brain metastasis	11.1%	14.3%	24.0%	
No brain metastasis	88.9%	85.7%	76.0%	
**Bone metastasis (**%**)**				0.795
Bone metastasis	24.1%	28.6%	20.0%	
No bone metastasis	75.9%	71.4%	80.0%	
**Liver metastasis (**%**)**				0.719
Liver metastasis	5.6%	9.5%	4.0%	
No liver metastasis	94.4%	90.5%	96.0%	
mPFS (months)	11.0	8.0	4.0	0.000*
mOS (months)	28.0*	20.0	23.0*	0.167
ORR	83.3%	76.2%	68.0%	0.302
DCR	94.4%	95.2%	92.0%	0.881

mPFS, Median progression-free survival; mOS, Median overall survival; ORR, Objective response rate; DCR, Disease control rate. *statistical significance at *p* < 0.05

The median PFS with TKI therapy in the three groups was 11 months (95% CI: 9.6–12.4) in the TPS < 1% group, 8 months (95% CI: 6.6–9.4) in the TPS 1–49% group, and 4 months (95% CI: 3.2–4.8) in the TPS ≥ 50% group, with statistically significant differences among the three groups (*p* = 0.000), as depicted in Fig. 2C. Using Cox regression analysis, both TPS 1–49% (HR 1.498, 95% CI: 1.187–2.581, *p* = 0.045) and TPS ≥ 50% (HR 2.760, 95% CI: 1.569–4.855, *p* = 0.000) groups significantly decreased the median PFS compared to the TPS < 1% group, as illustrated in Table 4; Fig. 4A.

**Table 4:** Associations of OS and PFS with PD-L1 expression

	HR (95% Cl) for PFS	*p*
**Primary lung site group**		0.931
TPS < 1%	1.00(ref.)	0.661
TPS 1 ∼ 49%	1.498(1.187, 2.581)	0.045*
TPS ≥ 50%	2.760(1.569, 4.855)	0.000*
**Lymph nodes group**		
TPS < 1%	1.00(ref.)	
TPS 1 ∼ 49%	1.116 (0.511, 1.912)	0.817
TPS ≥ 50%	1.081 (0.561, 2.083)	0.612
	HR(95%Cl)for OS	*p*
**Primary lung site group**		
TPS < 1%	1.00(ref.)	
TPS 1 ∼ 49%	1.444(0.671, 3.108)	0.389
TPS ≥ 50%	2.126(1.044, 4.332)	0.038*
**Lymph nodes group**		
TPS < 1%	1.00(ref.)	
TPS 1 ∼ 49%	0.960(0.380, 2.425)	0.931
TPS ≥ 50%	1.785(0.758, 4.205)	0.185

Data presented as hazard ratio (95% confidence interval). *statistical significance at *p* < 0.05

The median OS of the three groups was 28 months (95% CI: 24.7–31.3) in the TPS < 1% group, 20 months (95% CI: 13.0–27.0) in the TPS 1–49% group, and 23 months (95% CI: 12.4–33.6) in the TPS ≥ 50% group, and the median OS of TPS < 1% and TPS ≥ 50% groups indicated a statistically significant difference (*p* = 0.008), as illustrated in Fig. 2D. The TPS ≥ 50% group had a lower median OS than the TPS < 1% group (HR 2.126, 95% CI: 1.044–4.332, *p* = 0.038). The TPS 1–49% group did not affect OS compared to the other groups, as depicted in Table 4; Fig. 4B.

These data support the predictive value of PD-L1 from the primary lung site for treating advanced EGFR-mutant NSCLC with EGFR-TKI.

### PD-L1 in the lymph nodes of EGFR-mutant patients was not associated with PFS and OS with EGFR-TKI therapy

According to PD-L1 expression in the metastatic lymph nodes, 84 patients were divided into TPS < 1%, TPS 1–49%, and TPS ≥ 50% groups. The characteristics of the patients are revealed in Table 5. The ORR of the three groups was 72.0% in the TPS < 1% group, 75.0% in the TPS 1–49%, and 77.8% in the TPS ≥ 50% groups. The DCR of the three groups was 88.0% in the TPS < 1%, 87.5% in the TPS 1–49%, and 88.9% in the TPS ≥ 50% groups. No statistical difference was observed in ORR (*p* = 0.886) and DCR (*p* = 0.992) among the three groups, as demonstrated in Fig. 3.

**Table 5:** Characteristics of patients in the lymph node group

	TPS < 1% (*n* = 50)	TPS 1 ∼ 49% (*n* = 16)	TPS ≥ 50% (*n* = 18)	*p*
Age (median)	60.0	54.0	67.0	0.037*
**Smoking history (**%**)**				0.262
Smoker	24.0%	25.0%	44.4%	
Non-smoker	76.0%	75.0%	55.6%	
**EGFR mutation (**%**)**				0.091
19del	48.0%	62.5%	55.6%	
21L858	52.0%	37.5%	44.4%	
**TNM stage (**%**)**				0.979
IIIB	20.0%	25.0%	22.2%	
IIIC	16.0%	12.5%	11.1%	
IV	64.0%	62.5%	66.7%	
**Brain metastasis (**%**)**				0.091
Brain metastasis	16.0%	0.0%	11.1%	
No brain metastasis	84.0%	100.0%	88.9%	
**Bone metastasis (**%**)**				0.690
Bone metastasis	32.0%	25.0%	22.2%	
No bone metastasis	68.0%	75.0%	77.8%	
**Liver metastasis (**%**)**				0.111
Liver metastasis	12.0%	0.0%	0.0%	
No liver metastasis	88.0%	100.0%	100.0%	
mPFS (months)	7.0	6.0	5.0	0.834
mOS (months)	29.0	24.0	20.0	0.344
ORR	72.0%	75.0%	77.8%	0.886
DCR	88.0%	87.5%	88.9%	0.992

mPFS, Median progression-free survival; mOS, Median overall survival; values are mean (SD) unless otherwise noted. *statistical significance at *P* < 0.05

The median PFS with TKI therapy in the three groups was 7 months (95% CI: 4.5–9.5) in the TPS < 1% group, 6 months (95% CI: 4.2–7.8) in the TPS 1–49% group, and 5 months (95% CI: 3.6–6.4) in the TPS ≥ 50% group. No significant correlation was identified between PD-L1 expression in lymph nodes and PFS among the three groups (Table 4; Fig. 4A).

The median OS of the three groups was 29 months (95% Cl: 19.6–38.4) in the TPS < 1% group, 24 months (95% CI: 15.8–32.2) in the TPS 1–49% group, and 20 months (95% CI: 11.9–28.1) in the TPS ≥ 50% group, without statistically significant difference among the three groups, as revealed in Fig. 2F. Similarly, no statistical differences existed between PD-L1 expression in lymph nodes and OS among the three groups (Table 4; Fig. 4B).

These data suggest that PD-L1 from metastatic lymph nodes may not be a reliable predictive marker for advanced EGFR-mutant NSCLC with EGFR-TKI therapy.

### Relationship between co-mutations and PD-L1 expression

Out of 184 patients, 41 underwent genetic testing using Next-Generation Sequencing (NGS). Among them, 56.1% harbored TP53 mutations, 19.5% had PIK3CA mutations, and 12.2% had NF1 mutations, with the remaining mutations collectively accounting for less than 10%. Within the TP53 mutation group, 52.2% (*n* = 12) had Tumor Proportion Score (TPS) < 1%, 21.7% (*n* = 5) had TPS 1 ∼ 49%, and 26.1% (*n* = 6) had TPS ≥ 50%. In the PIK3CA mutation group, TPS < 1% accounted for 62.5% (*n* = 5), TPS 1 ∼ 49% for 12.5% (*n* = 1), and TPS ≥ 50% for 25.0% (*n* = 2). In the NF1 mutation group, TPS < 1% accounted for 60.0% (*n* = 3), TPS 1 ∼ 49% for 20.0% (*n* = 1), and TPS ≥ 50% for 20.0% (*n* = 1). According to our statistical analysis, TP53 mutation (*p* = 0.438), PIK3CA mutation (*p* = 0.149), and NF1 mutation (*p* = 0.258) had no significant impact on PD-L1 expression, as illustrated in Table S1.

### Discussion

This study retrospectively analyzed the relationship between PD-L1 expression in primary lung site or metastatic lymph nodes and the prognosis of advanced EGFR-mutated NSCLC patients treated with first-generation EGFR-TKIs. High PD-L1 expression in the primary lung site was linked to shorter PFS and OS after EGFR-TKI therapy. In contrast, PD-L1 expression in metastatic lymph nodes was not significantly correlated with the prognosis of EGFR-mutant patients after EGFR-TKI therapy.

Evidence suggests that PD-L1 expression is lower in patients with EGFR-mutated NSCLC than in EGFR-wild-type NSCLC [16, 20, 25, 26]. The relationship between PD-L1 expression and the efficacy of TKI in patients with EGFR-mutated has demonstrated controversial findings in different studies. Some studies have found that PD-L1 expression was unrelated to EGFR-TKI efficacy [20, 27]. In contrast, other studies have suggested that high PD-L1 expression in patients with EGFR-mutated predicts better treatment outcomes after receiving EGFR-TKI [19, 21]. Nevertheless, more studies revealed that high PD-L1 expression predicted poor efficacy for patients with EGFR-mutated after TKI therapy [7, 14–18]. PD-L1 contributes to primary resistance to EGFR-TKI in EGFR-mutant NSCLC cells, which may be mediated through EMT induction via the activation of TGF-β/Smad canonical signaling pathway [28]. Our study found that the higher the expression of PD-L1 in the primary lung site of NSCLC patients with EGFR mutation, the lower the benefit achieved in PFS and OS after receiving first-generation EGFR-TKI. However, no correlation existed between PD-L1 expression and OS when the primary lung and metastatic lymph node sampling sites were included. Taking the above data together, whether the expression of PD-L1 in patients with EGFR mutations can predict the efficacy of EGFR-TKI is controversial. The conflicting conclusions in different studies include the relatively small sample sizes, different races, dynamic changes in PD-L1 after treatment, different studies that have used non-standardized methods for assessing PD-L1 expression, and different PD-L1 antibodies [15]. All patients in our study are treat-naive patients of Asian. PD-L1 testing was performed using 22C3 and DACO platforms, all adjudicated by pathologists from a single medical center. Given these considerations, the bias of assessment is likely minimal. After accounting for the correlation between PD-L1 and survival, which differed when including samples from the metastatic lymph nodes, we consider that the spatial heterogeneity of PD-L1 expression may lead to different conclusions in different studies.

PD-L1 expression varies substantially across different anatomical sites [23]. Previous literature found that PD-L1 expression in metastatic lymph nodes was usually higher than in primary lung sites [24, 29, 30]. Therefore, PD-L1 from metastatic lymph nodes was an unreliable predictive marker for immunotherapy treatment of advanced driver-negative NSCLC [29]. Previous research has paid little attention to the correlation between PD-L1 expression in metastatic lymph nodes and the prognosis of TKI therapy in NSCLC patients with EGFR mutations. Our study found that despite no significant difference between PD-L1 expression in metastatic lymph nodes and primary lung sites in EGFR-mutant NSCLC, PD-L1 expression in metastatic lymph nodes did not affect PFS and OS of the first-generation TKI treatment, contrary to the predictive value of PD-L1 expression in primary lung site.

PD-L1 expression in NSCLC is influenced by various factors, including tumor hypoxia, a pro-inflammatory, interferon-gamma-rich microenvironment, as well as the activation of numerous intracellular pathways that promote cell motility and survival, including phosphatydil-inositol-3-kinase/AKT and Ras-Raf-Erk pathways [31]. The mechanism of PD-L1 upregulation in metastatic lymph nodes may differ from that of primary ones because lymph nodes are more enriched with tumor-infiltrating lymphocytes. The interaction of lymphocytes with the tumor cell mesenchyme can cause upregulation of PD-L1 expression [32] or cause PD-L1 upregulation through the activation of T-cells to produce IFN-γ [33]. Consequently, PD-L1 expression in the primary instead of elevated PD-L1 from lymph nodes may be more predictive of the therapeutic efficacy of TKI. These findings suggest that biopsies from the primary rather than lymph nodes are preferred for PD-L1 testing to predict TKI values in clinical practice. Interestingly, data from the FMI database on triple-negative breast cancer demonstrated that PD-L1 positivity rates were significantly lower in metastatic lesions than in primary tumors [34], in contrast to our results for lung cancer with EGFR mutations. However, in the above study on breast cancer, PD-L1 expression was scored on immune cells instead of cancer cells.

In addition, a previous study showed significant deviations in PD-L1 expression according to the histologic subtype. Patients with adenocarcinomas were more likely to have high PD-L1 expression in metastatic sites than in primary site, contrary to the results for patients with squamous histology [24]. However, the above study did not address whether the patients harbored EGFR. Most patients included in the study were Caucasian, exhibiting a low mutation rate of EGFR, implying that the above conclusion might apply to EGFR-negative populations. All patients with EGFR mutations were included in this study. However, the results revealed no significant difference in PD-L1 expression between metastatic lymph nodes and the primary lung site, inconsistent with previous studies. This may be because EGFR mutation status varies substantially among patients from one study to another.

This study has some limitations. First, because this was a retrospective study, these intriguing findings should be interpreted cautiously. Second, no pairwise comparisons of patients with the primary and lymph nodes were performed. Finally, third-generation TKI, such as osimertinib, has become more commonly employed, and the results should be validated in patients treated with third-generation EGFR-TKI. Accordingly, further studies, ideally on larger prospective cohorts, are warranted to address these critical interactions between PD-L1 expression at different sites and the prognosis of EGFR-TKI.

### Conclusion

In conclusion, this study found that PFS and OS were shorter in advanced EGFR-mutant NSCLC patients with high PD-L1 expression in the primary lung site after TKI therapy. PD-L1 expression in primary tumors may serve as a predictive biomarker of survival for first-generation EGFR-TKI therapy. However, PD-L1 expression in lymph nodes was unrelated to either PFS or OS in patients receiving EFGR-TKI therapy. PD-L1 from different biopsy sites may have different predictive values for the benefit of first-generation EGFR-TKIs in advanced EGFR-mutant NSCLC.

### Electronic supplementary material

Below is the link to the electronic supplementary material.

Supplementary Material 1

## Data Availability

All presented data in this study are available from the corresponding author upon reasonable request.

## Abbreviations

NSCLC: Non-small cell lung cancer
LUAD: Lung adenocarcinoma
EGFR: Epidermal growth factor receptor
TKI: Tyrosine kinase inhibitor
PD-L1: Programmed cell death ligand 1
TNM: Tumor, node, metastasis
MRI: Magnetic resonance imaging
ECT: Emission computed tomography
RECIST: Response Evaluation Criteria in Solid Tumors
CR: Complete response
PR: Partial response
SD: Stable disease
PFS: Progression-free survival
OS: Overall survival
ORR: Objective response rate
DCR: Disease control rate
ARMS: Amplification refractory mutation system
NGS: Next-generation sequencing
TPS: Tumor Cell proportion Score
TBB: Transbronchial biopsy
TBLB: Transbronchial lung biopsy
TBNA: Transbronchial needle aspiration
PS: Performance status
LN: Lymphe node
