# Supplementary material for: Heterogeneity in PD-L1 expression between primary and metastatic lymph nodes: a predictor of EGFR-TKI therapy response in non-small cell lung cancer
Source: Respir Res. 2024 Jun 5;25:233. doi: 10.1186/s12931-024-02858-3 (PMC11151486; doi:10.1186/s12931-024-02858-3)
Supplement: Supplementary file 1 — Supplementary Material 1 [file 12931_2024_2858_MOESM1_ESM.docx]

Table S1. Prevalent Co-Mutations (>10%) Identified in 41 Patients via NGS

|  | TPS<1% | TPS 1~49% | TPS≥50% | *p* |
| --- | --- | --- | --- | --- |
| TP53(%) |  |  |  | 0.438 |
| Positive | 52.2% | 21.7% | 26.1% |  |
| Negative | 47.8% | 78.3% | 73.9% |  |
| PIK3CA |  |  |  | 0.149 |
| Positive | 62.5% | 12.5% | 25% |  |
| Negative | 37.5% | 87.5% | 75% |  |
| NF1 | 18.5% | 33.3% | 20.0% | 0.258 |
| Positive | 60% | 20% | 20% |  |
| Negative | 40% | 80% | 80% |  |

**NGS**, Next-Generation Sequencing
